# New insights into the role of soluble E-cadherin in tumor angiogenesis

**DOI:** 10.15698/cst2018.09.154

**Published:** 2018-08-20

**Authors:** Maggie K.S. Tang, Philip P. Ip, Alice S.T. Wong

**Affiliations:** 1 School of Biological Sciences, University of Hong Kong, Pokfulam Road, Hong Kong.; 2 Department of Pathology, University of Hong Kong, Pokfulam Road, Hong Kong.

**Keywords:** soluble E-cadherin, ovarian carcinoma, angiogenesis, exosome, β-catenin, NFκB

## Abstract

A key to successful metastasis is the formation of new vasculature, known as angiogenesis. Therefore, it is of great interest to unravel the underlying molecular mechanisms of tumor angiogenesis. Cadherins are a major class of cell surface receptors. The loss of cadherins, especially E-cadherin, is a well-established marker for tumor metastasis. Loss of E-cadherin is also a defining characteristic of several carcinomas, such as lobular carcinoma of the breast, and de-differentiated endometrioid carcinoma of the endometrium and ovary, which are known to be associated with more aggressive tumor behavior. Although E-cadherin is synthesized as a transmembrane molecule, its extracellular domain can be enzymatically cleaved off and released as a soluble E-cadherin (sE-cad), and this accounts for the loss of E-cadherin function or expression that has been implicated in tumor progression and metastasis. Importantly, sE-cad is present at high levels in the serum and malignant ascites of ovarian carcinoma patients. Nevertheless, little is known about how this essential protein dictates metastasis. Hitherto, many studies have given attention only to the dominant negative role of the loss of E-cadherin in weakening cell-cell adhesion, however, it is not known if sE-cad has biological activity in itself. In addition, the release mechanism of sE-cad has remained elusive. Here we show for the first time that sE-cad is a pivotal regulator of angiogenesis. We further show that exosomes are a novel major platform for the cleavage and release of sE-cad *in vitro*, *in vivo* and in patients’ derived samples (Nat Commun, 9: 2270).

Ovarian cancer, the leading cause of death among all gynecological malignancies, is a highly aggressive disease with a propensity for metastasis. Due to the discreet anatomical location, by the time patients become symptomatic, they already have advanced stage disease. Metastases are usually characterized by widespread peritoneal dissemination and development of malignant ascites. The metastatic progression of ovarian cancer involves dynamic changes in cell-cell adhesion. E-cadherin, being the best characterized cell-cell adhesion molecule, plays a crucial role in mediating cell-cell contacts at the adherens junctions. Although E-cadherin was originally described as being exclusively a transmembrane molecule, there are recently published data describing an 80 kDa soluble form of E-cadherin (sE-cad). However, previous studies mainly focused on the role of sE-cad in weakening cell-cell adhesion. It is still not known whether sE-cad has biological functions in itself. Given the presence of high levels of sE-cad in malignant ascites, the most distressing complication of ovarian cancer, we questioned if sE-cad could affect angiogenesis, which is closely associated with the formation of malignant ascites characteristic of tumor aggressiveness and metastatic potential. Our findings are the first to demonstrate sE-cad’s role in the pathogenesis of malignant ascites and reveal a new direction for understanding the oncogenic roles of sE-cad.

Angiogenesis is a multistep process which involves cell adhesion, growth, migration, and differentiation. As such, we assayed for these activities using various *in vitro* and *in vivo* approaches such as migration assay, tube formation assay, permeability assay and Matrigel plug assay to define a mechanism of action. We have shown for the first time that sE-cad is a key regulator of tumor angiogenesis, which is involved in one or more rate-limiting steps of the angiogenic cascade *in vitro* and *in vivo*. High sE-cad expression can be found in the conditioned media of ovarian cancer cell lines with high metastatic potential as compared to normal ovarian surface epithelial cells and fallopian tube epithelial cells. With an E-cadherin neutralizing antibody, HECD-1, we demonstrated the angiogenic function of sE-cad, but not other cadherins, both *in vitro* and *in vivo*. While both vascular endothelial growth factor (VEGF) and sE-cad are important contributors to angiogenesis, they act via independent pathways. Given that VEGF-based therapies often face the development of resistance and can also interfere with the normal VEGF pathway (e.g. the VEGF-inhibitor Bevacizumab), which leads to numerous adverse side effects, it is of great interest to identify other angiogenic regulators, such as sE-cad, with complimentary mechanisms.

Besides ectodomain shedding, we detected a predominant localization of E-cadherin in the Golgi/Trans-Golgi network using a refined analysis of sucrose gradient subcellular fractionation. This raises the possibility that E-cadherin can be released in microvesicles. By employing various tests on morphology (electron microscope; disc-shaped), density (sucrose gradient; 1.1036 - 1.1612 g/cm^3^), protein composition (Western blotting; Hsp70, CD63 and Tsg101), and size (nanoparticle tracking; 50 - 150 nm), we showed exosomes as a vehicle for sE-cad. Using immunoelectron microscopy and BODIPY-TR ceramide fluorescent dye, we further showed the surface localization and uptake of exogenous purified sE-cad-positives exosomes by endothelial cells. Surface localization of sE-cad will allow easy access for targeting neutralizing antibodies, thus effective functional blocking could be achieved as a potential therapeutic strategy. However, whether the uptake is mediated through an active targeting or passive random diffusion still awaits further investigation. sE-cad-positive exosomes are a potent inducer of angiogenesis. When injected into NOD/SCID mice preinoculated with ovarian cancer cells, sE-cad-positive exosomes significantly promoted cancer metastasis and ascites formation. Furthermore, sE-cad-positive exosomes could also be found in ascites of ovarian cancer patients and promoted angiogenesis *in vitro* and *in vivo*, confirming that the involvement of sE-cad is clinically relevant. The presence of sE-cad-positive exosomes in patients of colon, breast, and liver cancers further suggests that our findings may have a broader implication in other tumor types. Constitutive expression of sE-cad-positive exosomes is significantly more prevalent in the ascitic fluid of ovarian cancer patients than in the peritoneal fluids of non-cancerous patients with benign tumors. While our data suggest an unfavorable survival outcome; we were not able to demonstrate a clearly significant prognostic correlation between high levels of sE-cad-positive exosomes and either histological subtypes or FIGO stages. This may be related to our relatively small sample size for each histologic subtype, samples being collected at different time point of the disease, different sizes of the primary or metastatic tumors, and variability in the extent and location of the metastases. Currently available databases are mostly based on tissue expression of target proteins. A database of exosome secretion of specific protein is still lacking. Such database will surely help to address limitations of the individual dataset in specific situation and to drive high quality clinical outcome analysis of these increasingly important nanovesicles.

In search of the mechanism underlying sE-cad regulation of angiogenesis, we found a previously uncharacterized heterodimerization of E-cadherin with VE-cadherin, representing a new and attractive mechanism whereby endothelial cells, which lack E-cadherin, can lead to angiogenic signaling. This heterophilic interaction may serve as a new paradigm of cell-cell communication. Downstream of this heterophilic transconnection is the activation of β-catenin and nuclear factor-κB (NFκB) signaling cascade. While a crosstalk between β-catenin and NFκB has been previously suggested, we demostrated a sequential activation of these two signaling pathways. The activation of β-catenin took place as early as 30 min upon treatment, whereas that of NFκB could only be detected 120 min post-treatment. This sequential activation may allow a sustained cellular response (**Figure 1**). We further demonstrated the nuclear translocation of β-catenin and NFκB subunits, suggesting the activation of these two pathways, and additionally corroborated their requirement as siRNA-mediated silencing of β-catenin and NFκB abolished sE-cad-mediated angiogenic effects. Since β-catenin is also known to be present in the exosome cargos and may get transferred to the endothelial cells, our data could not distinguish the origin of the observed nuclear accumulation of β-catenin. Several questions could be raised from our findings. First, exosomes are involved in various functions in tumor growth and progression including angiogenesis, chemoresistance, extracellular matrix remodeling, and tumor immune escape. However, it is still unclear if there are subpopulations of exosomes each with a different and specific function as suggested by a wide variety in exosome morphology or a single exosome with multiple functions as hinted at by the diversified cargos in a single exosome. Second, we have only tested the effect of sE-cad-positive exosomes on endothelial cells; whether these exosomes will react with other components in the tumor microenvironment, such as stromal, mesothelial and immune cells to mediate other cellular responses will await further investigations. Third, while differential centrifugation and salting out are the standard practice for isolating exosomes, a mixed population of vesicles is unavoidable. Finally, exosomes are undoubtedly a promising therapeutic target. Recent studies have demonstrated that tumor-derived exosomes were composed of mixed subpopulations with specializations. While some play pro-tumor roles such as chemoresistance, angiogenesis and microenvironment remodeling, other could mediate anti-tumor immune response. These opposing functions suggest that one should be very precise when designing exosomes-based cancer therapy, and specific targeting a particular population of exosomes of a heterogeneous pool is essential. A high quality isolation method, followed by analysis of the contents, and *in vivo* tracking of the exosomes are essential not only to dissect the biological functions and transport of exosomes but also may allow more practical applications in diagnostics and therapeutics development.

**Figure 1:**
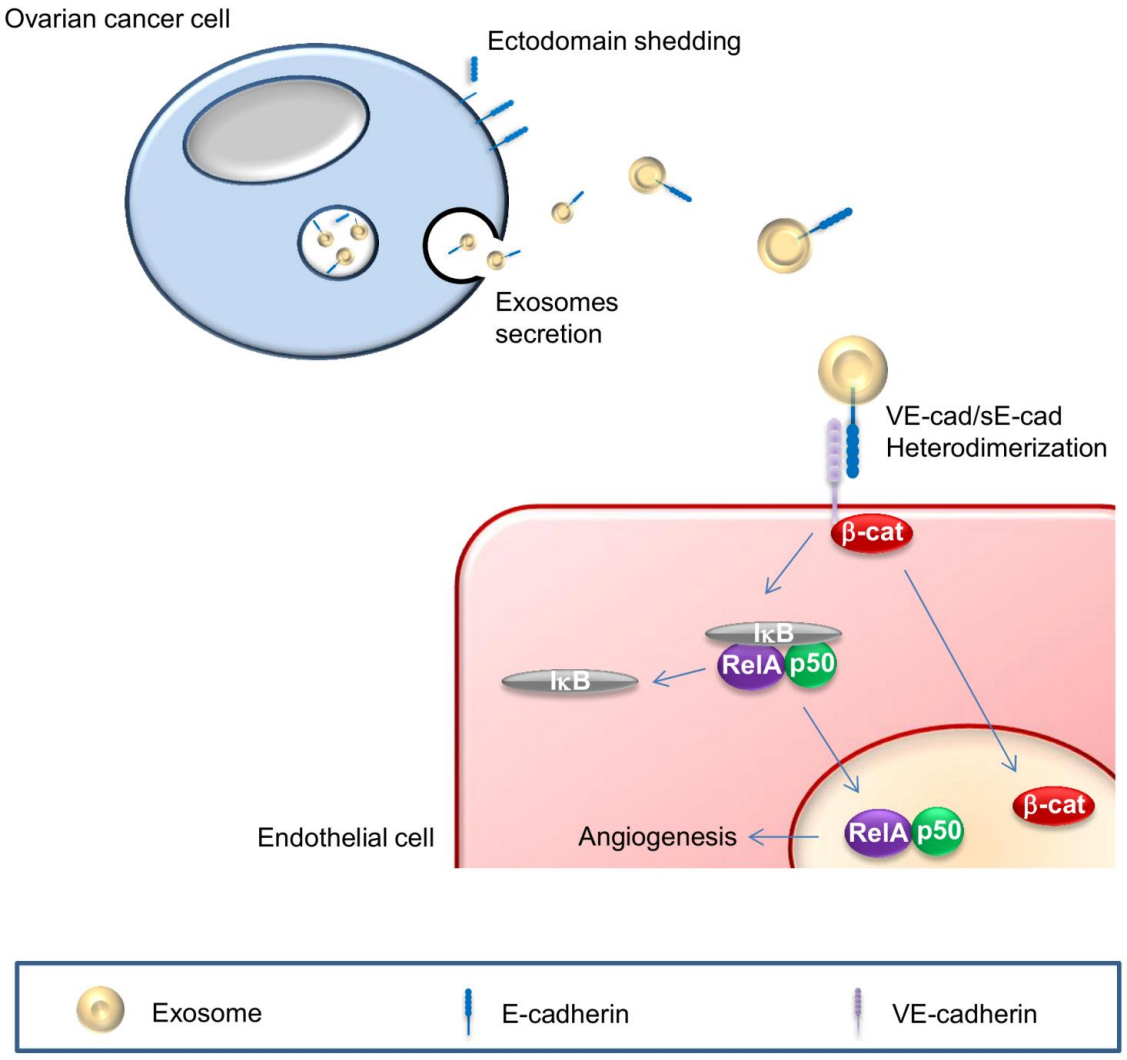
FIGURE 1: Schematic of sE-cad-positive exosomes in angiogenesis. sE-cad localized on exosome surface heterodimerizes with VE-cadherin and mediates a sequential activation of β-catenin and NFκB signaling cascades.

